# Defining control reference ranges in biologic samples in analytical laboratories

**DOI:** 10.3389/fpubh.2025.1618114

**Published:** 2025-08-28

**Authors:** Bart Vrugt, Barbara K. Kuhn, Richard Attanoos

**Affiliations:** ^1^Silag—Swiss Laboratory for Particle Analysis in Tissues, Zürich, Switzerland; ^2^Institute of Pathology, Stadtspital Triemli, Zürich, Switzerland; ^3^Department of Cellular Pathology, School of Medicine, Cardiff University, University Hospital of Wales, Cardiff, Wales, United Kingdom

**Keywords:** control subjects, asbestos, fiber analysis, tissue burden, asbestosis range

## Abstract

Mineral analytic laboratories define control reference ranges to interpret the significance of an individual’s prior exposures. Control reference ranges are internally compiled and defined for two scenarios: background controls—only subjects with ambient asbestos exposure and no increased risk of asbestos related disease, and asbestosis range controls, utilized for the diagnosis of asbestos-related lung fibrosis/asbestosis and for asbestos-related lung cancer causation. The objective of this study was to evaluate how different analytic laboratories have established their internal control reference ranges and to comment on their significance. The study comprised a review of the scientific literature generated from a Pubmed search of mineral analytic data from lung tissue in laboratories determining background exposures to asbestos and other elongate minerals. Twenty-six publications were found from 17 laboratories across Europe, North America, and Asia which had internally defined background control populations. The studies showed marked heterogeneity having been conducted over decades, using different criteria, different microscopic methodologies, and assessment of different fiber dimension. The most common criterion to define background control subjects was to establish individuals with no known occupational history of asbestos exposure and/or no evidence of asbestos-related diseases. In background controls with no disease, chrysotile was reported most frequently. Chrysotile and amphiboles were variably detected in lung tissue from control subjects in virtually all studies. Interlaboratory variations exist so individual results obtained in one laboratory do not transfer significance to another laboratory. The use of negative control groups in case–control studies is discussed alongside their relevance in ensuring the validity of results related to asbestos exposure and its diseases.

## 1 Introduction

Asbestos exposure is a recognized risk factor for a variety of serious diseases, including asbestosis, lung cancer and mesothelioma. Research on the mechanisms underlying these diseases, as well as preventive measures and therapies, requires precise studies and the use of appropriate control groups. Asbestos fiber concentration in human lung tissue has proved to be an important biomarker for asbestos-associated lung diseases (1, 2). Since Wagner (3) reported 33 cases of diffuse pleural mesothelioma in the Northwest Province following exposure to crocidolite asbestos, there has been a myriad of analytic epidemiologic studies correlating asbestos and disease in worker cohorts, and case–control studies which have demonstrated the importance of asbestos fiber type, fiber size, fiber biopersistence and latency in the induction of disease. Correlative mineralogic and pathologic studies (4–7) have played a key role in complimenting analytic epidemiology. These mineral analyses have consistently identified that mesothelioma and lung cancer risk, as well as extent of lung fibrosis/asbestosis is correlated with the retained elevated amphibole asbestos fiber content. These studies have also demonstrated that there is no correlation between asbestos related disease and retained chrysotile asbestos, a known biosoluble mineral in human tissue. Mineral analysis has provided complimentary evidence that chrysotile exposure is far less potent than commercial amphibole (crocidolite and amosite) asbestos in inducing asbestos-related diseases. In this analysis, the role of establishing control reference populations is a key tool to contextualizing the significance of an exposure to a mineral.

Control subjects are basically defined by the absence of any known occupational, para-occupational and/or environmental exposure to asbestos, as well as an absence of any asbestos-related disease. In asbestos-related disease risk-assessment, a negative control group serves as a baseline reference to define thresholds reflecting ambient air exposure. There is no significant association between ambient asbestos exposures and the development of asbestos related disease (8–13). In addition, it is recognized that ambient air asbestos fiber concentrations may vary over 10-fold in different geographic locations without any impact on the development of mesothelioma (12). With respect to control reference ranges—these are established within individual analytical laboratories performing fiber burden analyses and the significance of an individual exposure may then be determined by benchmarking the case to the established laboratory control population (14–20). The control population represents, most closely, only subjects with ambient asbestos exposure and no increased risk of asbestos related disease (21). The scientific evidence correlating cumulative asbestos exposure with disease has been extensively established in occupational settings which are many orders of magnitude above background ambient exposure levels. Numerous analytical laboratories have made correlations between retained amphibole asbestos fiber concentrations in lungs of asbestos exposed workers and asbestos related disease (4, 6, 22).

The Helsinki criteria proposed (23) the use of two sets of controls: a “background” control reference population for subjects without known significant exposure or disease and a control named “asbestosis range” reflecting the retained amphibole asbestos fiber count for cases with established histologic asbestosis. This background control can be used in determining causality for mesothelioma. The asbestosis range control can be utilized in claimed asbestos exposed subjects with suspected lung fibrosis, to allow for a determination of disease diagnosis—asbestosis, and for disease causality in asbestos-related lung cancer *ex asbestosis*. The proposal by the College of American Pathologists-Pulmonary Pathology Asbestosis Guidelines Committee (24) highlighted the use of the total amphibole asbestos count without consideration of chrysotile and the 5th percentile value of an asbestosis cohort as the “lower asbestosis range” value for use in analytic laboratories. These proposals were accepted by the Helsinki update (25).

Studies using lung tissue fiber analysis have shown an increased burden of amphibole asbestos in lungs of patients with asbestosis and mesothelioma compared to non-exposed individuals. Since the ninety’s the use of asbestos has been banned in western countries. Consequently, lung tissue fiber content in patients with mesothelioma has gradually dropped to levels generally observed in lungs of non-exposed individuals (26, 27) suggesting that other mechanisms unrelated to asbestos, such as exposure to other particles or genetic predisposition, have become more relevant in the carcinogenesis of mesothelioma (21, 28–33). This also stresses the need for universal criteria and technical standardization for detection of asbestos fibers to better identify negative control subjects with lung tissue fiber content that reflects ambient air exposure.

## 2 Methods

**Figure 1:**
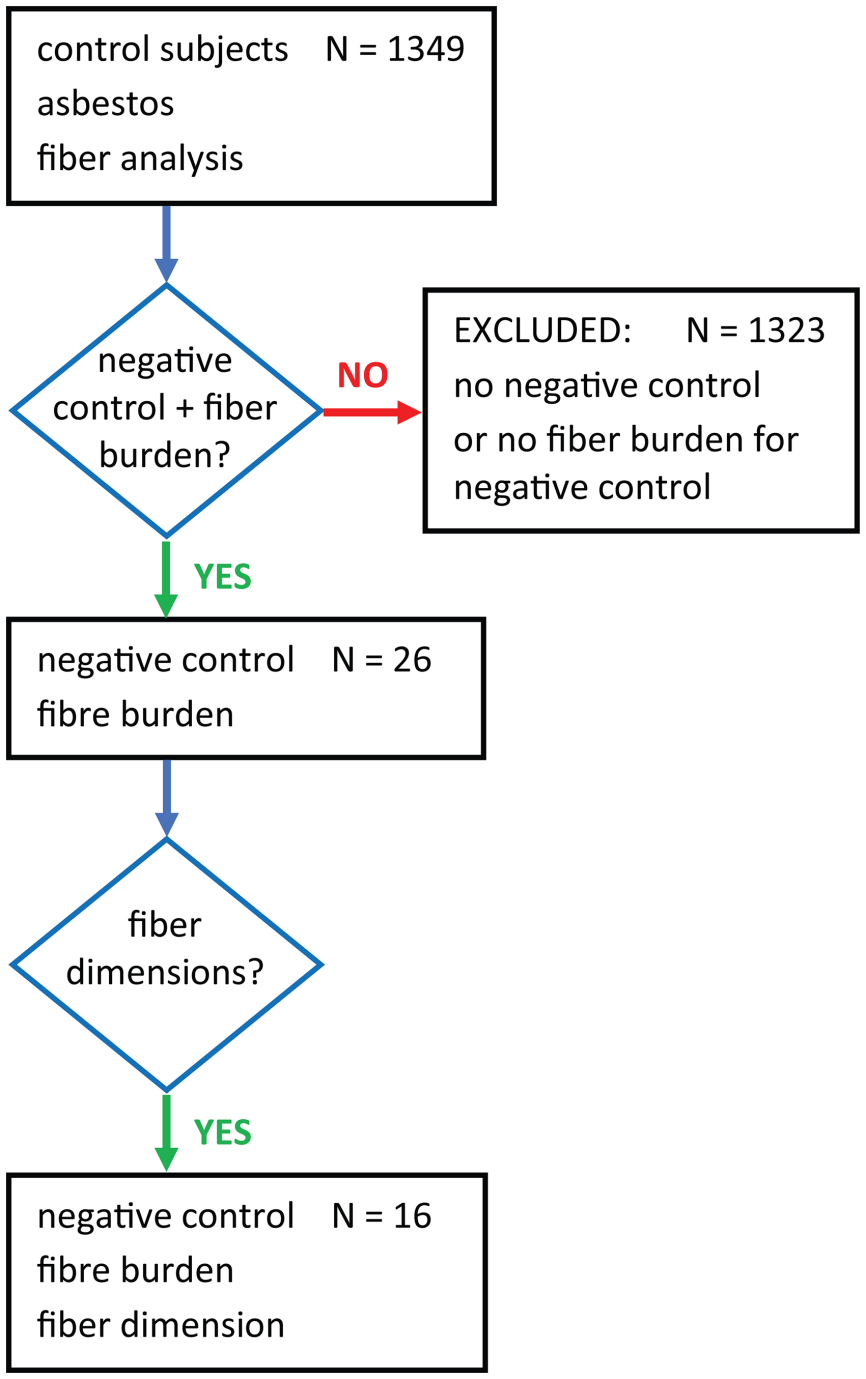
Flow chart showing the terms used for the PubMed search and subsequent filtering for negative controls with fiber burden data.

We aimed to gather information on asbestos content in lung tissue of non-exposed subjects. There are various terms to describe such a population: background or negative control, exposure to ambient air. Therefore, we performed a PubMed search using the general terms *control subjects*, *asbestos* and *fiber analysis*. Of the 1,349 hits, 26 papers (1, 15–18, 22, 26, 34–51) were selected for this review as they contained comprehensive information on negative control groups including fiber burden summarized in the flow chart in Figure 1. 1,323 publications studying asbestos fiber content only in occupationally exposed subjects and other tissues or air samples were excluded. For the selected studies, information on the selection criteria for control subjects, age, gender, occupational and environmental exposure, methods of fiber analysis, fiber type and fiber dimensions were collected for comparison.

## 3 Results

### 3.1 Selection criteria for control subjects

Twenty-six studies had been conducted in different Countries over a period of 46 years. There was observed marked heterogeneity in the analytical laboratory control data. This is an expected finding as the individual laboratories select non-standardized case series, incorporate varied subjects with different historic exposures, from different geographic settings and use different analytical methods. Data concerning Country, size of control groups, demographic data profile—age range, gender, inclusion criteria, claimed exposures, laboratory methodology, and unit used to report values are summarized in Table 1. Analytical parameters including fiber dimensional data, fiber burden, asbestos subtypes, are summarized in Table 2.

**Table 1:** Summary of 26 studies (1977–2023) that selected control subjects based on different criteria.

Study	Year	Country	Controls (M/F)	Age	Criteria	Exposure	Method	Unit used
Whitwell et al. (1)	1977	UK	100 (71/28)	>20	1 2	Possible	LM	dry
Churg and Warnock (49)	1980	USA	21 (11/10)	43–95	5* 2	1 of 21	TEM	wet
Churg (50)	1982	USA	20 (−/−)	-	2	-	TEM	mixed
Mowe et al. (51)	1985	Norway	28 (28/0)	42–83	1	21 of 28	SEM	dry
Roggli et al. (34)	1986	USA	20 (−/−)	28–85	1	-	SEM	wet
Case and Sebastien (35)	1987	Canada	23 (17/6)	75 ± 8	2	None	TEM	dry
Case and Sebastien (35)	1987	Canada	23 (17/6)	73 ± 8	2	Environmental	TEM	dry
Gaudichet et al. (36)	1988	France	20 (−/−)	-	3	Possible	TEM	dry
Albin et al. (37)	1990	Sweden	89 (−/−)	37–93	2	-	TEM	dry
Romer (38)	1993	CH	26 (14/12)	58–93	2 1	Possible	TEM	dry
Dawson et al. (39)	1993	UK	31 (0/31)	30–93	2 1	-	TEM	dry
Sakai et al. (40)	1994	Japan	16 (12/4)	43–78	1	Possible	TEM	dry
Srebo et al. (15)	1995	USA	19 (19/0)	28–85	2 1 7	-	SEM	wet
de Vuyst (16)	1998	Europe		-		-	TEM	dry
Dodson et al. (17)	1999	USA	33 (23/10)	12–73	2 5 1	Urban	TEM	dry
Dodson et al. (41)	2000	USA	21 (−/−)	-	2 1 5	-	TEM	dry
Roggli et al. (22)	2002	USA	19 (−/−)	-	7	-	SEM	wet
Roggli and Sharma (42)	2004	USA	-	-	7 5	-	TEM	wet
Gibbs et al. (18)	2005	UK	254 (233/21)	26–87	1 2	Urban, rural	TEM	dry
Roggli and Vollmer (26)	2008	USA	20 (−/−)	-	7 1 2 5	-	SEM	wet
Han et al. (43)	2009	S-Korea	36 (25/11)	14–83	4 2 1	-	TEM	dry
Gilham et al. (44)	2016	UK	262 (−/−)	-	2	-	TEM	dry
Velasco-Garcia et al. (45)	2017	Spain	5 (−/−)	48–78	1	Urban	SEM	dry
Gordon (46)	2019	USA	207 (−/−)	>35	2 6	Urban	TEM	wet
Kuhn (internal data)	2019	CH	11 (−/−)	-	2	-	TEM	dry
Schneider et al. (47)	2023	Germany	17 (−/−)	59.8 ± 10	2	-	SEM/TEM	dry
Visona et al. (48)	2023	Italy	50 (−/−)	72.3 ± 11.2	4 1 2	-	SEM	dry

Applied criteria are listed in the table in the order of importance starting with the most important criteria: (1) absence of asbestos-related diseases; (2) no occupational exposure; (3) cardiovascular disease; (4) violent death; (5) less than 20 asbestos bodies per gram wet tissue; (5)^*^: less than 100 AB per gram wet tissue; (6) exclusion when crocidolite or amosite was detected, and (7) macroscopically normal lung.

**Table 2:** Summary of 26 studies (1977–2023) continued giving analytical information and asbestos burden per gram dry tissue separated for different categories.

Study	Year	Fiber length μm	Reported values	AAF detected in controls	AB/g dry	AF/g dry	AAF/g dry	Chrys/g dry
Whitwell et al. (1)	1977	6	Range	-		50,000		
Churg and Warnock (49)	1980	1	Mean, range	tre, act, am, croc	840		750,000	6,800,000
Churg (50)	1982	-	Mean, range	tre, act, anth	280	1,290,000		
Mowe et al. (51)	1985	-	Range	-	840	4,800,000		
Roggli et al. (34)	1986	5	Range	-	220			
Case and Sebastien (35)	1987	5	Mean	tre, am, croc		260,000	120,000	80,000
Case and Sebastien (35)	1987	5	Mean	tre, am, croc		570,000	140,000	280,000
Gaudichet et al. (36)	1988	-	Mean	am, croc	1,000	4,000,000	800,000	
Albin et al. (37)	1990	-	Range	all but act				300,000,000
Romer (38)	1993	1	Range	-	3,000	300,000		
Dawson et al. (39)	1993	-	Mean, range	croc, am, tre			1,000,000	20,100,000
Sakai et al. (40)	1994	-	Range	croc, am		14,000,000	7,700,000	8,800,000
Srebo et al. (15)	1995	5	Range	tre, anth, act	220	127,000	25,400	10,000
de Vuyst (16)	1998	1	Maximum			4,000,000	2,000,000	
Dodson et al. (17)	1999	0.5	Range	all 5	200	290,000	290,000	210,000
Dodson et al. (41)	2000	0.5	Range	all 5	101	290,000		210,000
Roggli et al. (22)	2002	5	Range	-	220	25,400	25,400	
Roggli and Sharma (42)	2004	-	Range	-	140	251,000		
Gibbs et al. (18)	2005	-	Median	am, croc, tre			300,000	5,230,000
Roggli and Vollmer (26)	2008	-	Range	-	200			
Han et al. (43)	2009	0.2	Range	croc, tre, act, am		3,880,000	1,000,000	3,670,000
Gilham et al. (44)	2016	5	Maximum	-		100,000		
Velasco-Garcia et al. (45)	2017	-	Median	all 5	599	no data		
Gordon (46)	2019	-	Range	tre	10		3,450	300,000
Kuhn (internal data)	2019	0.5	Range	-	400	220,000		
Schneider et al. (47)	2023	5	Maximum	-	78 /cm^3^		140,000	180,000
Visona et al. (48)	2023	5	Mean	tre, anth, am	2,014	3,188		489

AB, asbestos bodies; AF, total asbestos fibers; AAF, amphibole asbestos fibers; Chrys, chrysotile asbestos fibers. Type of amphibole asbestos (AAF): amosite (am), crocidolite (croc), tremolite (tre), actinolite (act) and anthophyllite (anth).

A diversity of inclusion criteria was observed when defining the control group. Table 1 lists the inclusion criteria (presented in order of their importance). Twelve studies used only one criterion for the selection of control subjects and 13 studies used two or more criteria in combination. The absence of known asbestos-related diseases was used as a primary criterion in six studies and as a secondary criterion in eight studies. No documented occupational asbestos exposure was used as a primary criterion in 11 and as a secondary criterion in six studies. One study selected their control subjects from patients with cardiovascular disease and two studies from accidental deaths. An *asbestos body burden* of less than 100 per gram wet tissue was used as primary criterion in one study and an *asbestos body* burden of less than 20 per gram wet tissue as secondary criterion in four studies. Three studies included subjects into their control group if the lung tissue looked macroscopically normal, and one study used this as a secondary criterion. The mere detection of amosite or crocidolite during analysis led to subsequent exclusion of subjects in one study. Because genomic testing has only recently become available the potential significance of genomic variability and its impact in controls was not provided in any of the studies.

### 3.2 Subject profile—number, age, and gender

The number of control subjects among the different studies ranged considerably from 5 to 262 (median 23). In two studies the number of control subjects was not mentioned. Basically, all studies had tried to match their control subjects with the patient group according to age. Gender was mentioned in 13 studies only. The number of female control subjects was considerably lower in all studies. In three studies female controls subjects were excluded.

### 3.3 Possible exposure of control subjects

After analysis of lung tissue four studies mentioned “possible” exposure to asbestos in some of their control subjects, thereby implying some subjects with unidentified remote exposure to above background asbestos had not been excluded using their screening methods. These studies selected their control subjects from autopsy records with the criteria no asbestos-related disease or from a cardiovascular disease group. Occupational history was checked retrospectively but often incomplete. No action (exclusion) was taken apart from reporting possible occupational exposure. One study (40) confirmed the inclusion of individuals with occupational asbestos exposure within their control population. Eleven studies mentioned possible urban and/or rural exposure to asbestos. Case and Sebastien (35) distinguished two control groups, an environmentally exposed group from a mining area and one without known exposure from outside of mining areas.

### 3.4 Analytical methods

Different preparation methods were applied to extract asbestos bodies (AB) and fibers from peripheral lung tissue for quantification. Wet digestion was used by 18 studies, low temperature ashing in six studies and two studies combined digestion and ashing. Hence, results are given either in AB or fibers per gram wet (7/26) or dry (18/26) lung tissue. In 15 studies numbers of AB were assessed using light microscopy of digested lung tissue and expressed as number of AB/g of lung tissue. In three studies, quantification of ferruginous bodies was performed on iron-stained sections (AB/cm^2^).

The first study in 1977 (1) used light microscopy to determine asbestos fibers extracted from peripheral lung tissue. Since electron microscopy techniques are available, either scanning (SEM) or transmission electron microscopes (TEM) mostly combined with energy dispersive X-ray spectroscopy (EDX) are the methods of choice. Eighteen studies used TEM and seven used SEM. One study (47) used a combination of both methods.

Minimum fiber length used to count and report fibers varied from 0.2 μm to 6 μm. Although, many studies (*n* = 11) did not specify the minimum fiber length used. Minimum fiber length longer than 5 μm was most popular (8), followed by longer than 1 μm (3) and 0.5 μm (3), longer than 6 μm (1) and 0.2 μm (1) are rare.

### 3.5 Fiber numbers and asbestos fiber types

**Figure 2:**
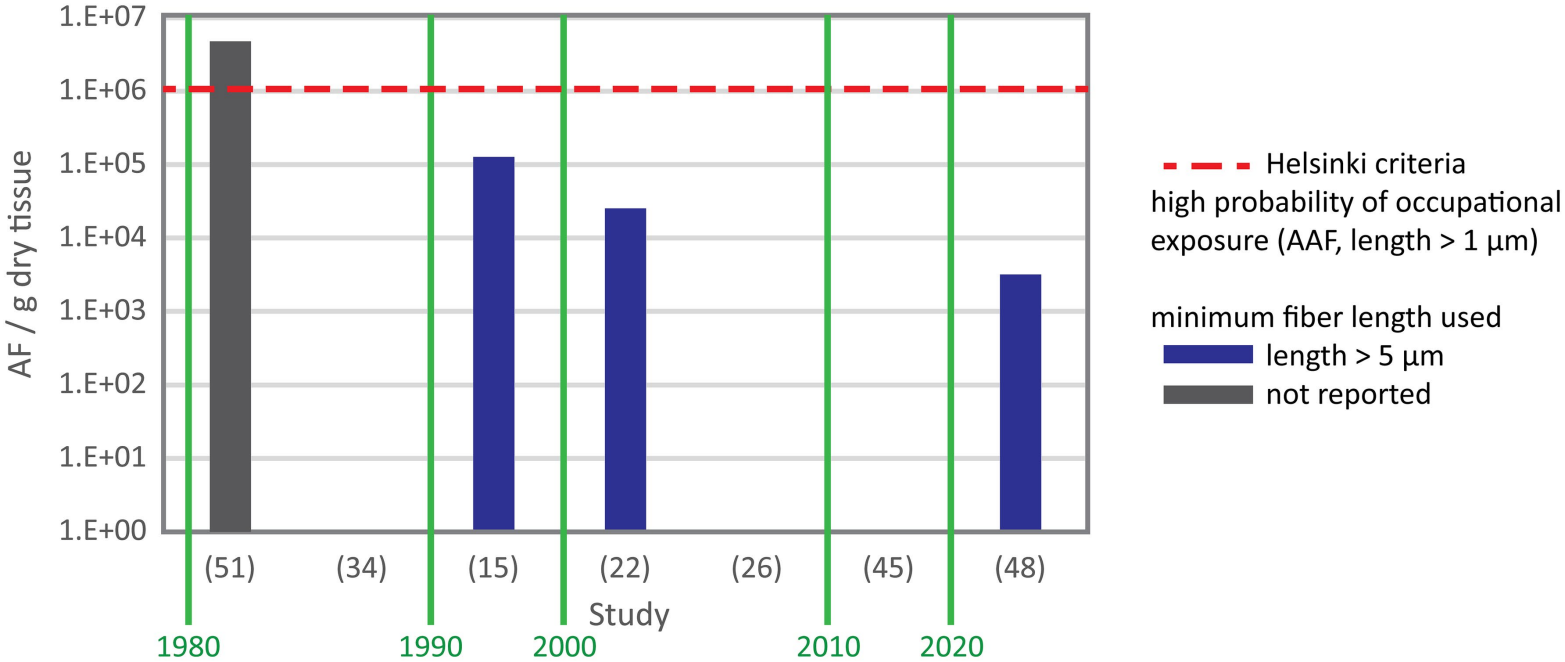
Total asbestos fiber content in negative control subjects from studies using SEM with study reference number on the X axis. Y axis gives the range except for (45) reporting the median and (48) reporting the mean. In study (51) fiber numbers exceeded the level seen in subjects with a possible occupational exposure. The authors confirmed that their control included individuals with occupational exposure. Study (48) used suboptimal setup of the SEM for fiber analysis and only reported the mean.

**Figure 3:**
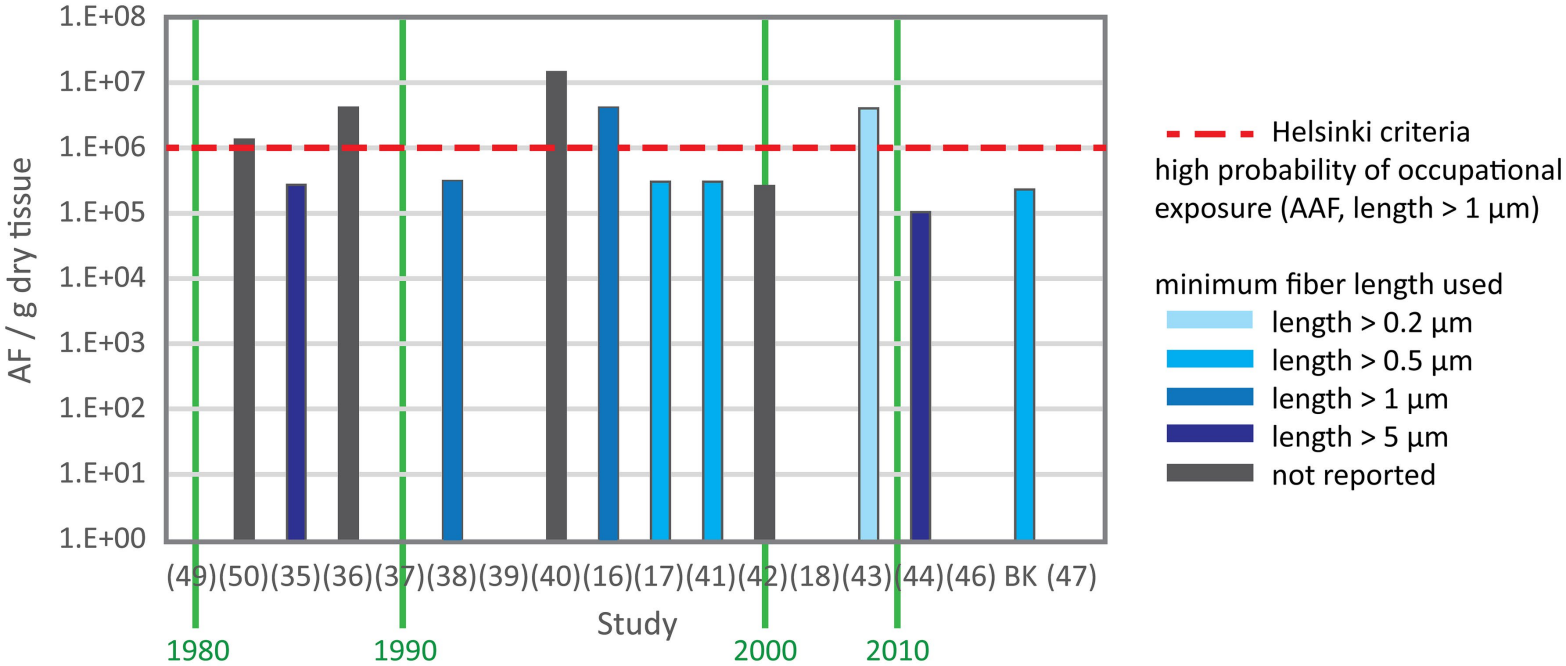
Total asbestos fiber content in control subjects from studies using TEM. X axis is the study reference number and Y axis is the range except for (35, 36, 50) reporting the mean and (18) reporting the median. A variety of minimum fiber length were used for fiber counting.

Asbestos content data were provided in number of total asbestos fibers, amphibole asbestos fibers or chrysotile fibers per gram lung tissue. Values were reported as mean, median or range in five, two and 17 studies, respectively. In three studies only maximum values were reported. Figure 2 shows the number of total asbestos fiber reported in studies using SEM for analysis. As most of these studies reporting fiber burden used 5 μm as minimum fiber length the data are comparable and show a decrease of asbestos content over three decades. Mowe et al. (51) reported fiber numbers in controls higher than the value indicating probable occupational exposure given by the Helsinki criteria (25) and confirmed also that several control subjects sustained possible occupational exposure. Figure 3 shows the total asbestos burden reported in studies using TEM for analysis. These fiber numbers cannot be compared as a variety of minimum fiber lengths were used for reporting.

Many control studies with subjects without disease report chrysotile as the most frequently detected asbestos fiber type. The amphibole asbestos fiber types are divided into two groups: amosite and crocidolite being the commercial amphibole asbestos types while asbestiform actinolite, anthophyllite and tremolite are the non-commercial amphibole asbestos types. Fourteen studies reported the occurrence of non-commercial amphibole asbestos in control subjects (Table 1). In 11 of these studies, amosite and crocidolite were also detected in control subjects.

## 4 Discussion

Twenty-six studies conducted in 12 countries in three continents and published over almost five decades (46 yrs) were systematically reviewed. Marked interlaboratory heterogeneity was observed which rendered comparative analysis between reported numeric controls across laboratories problematic. Interlaboratory variation can be attributed to differences in sample preparation, analytical methods and statistical analyses. Differences in the geological setting, the degree of urbanization and industrialization can lead to variations in background exposure of different populations adding to the interlaboratory variations. This finding does not, in any way, diminish the significance or worth of mineral analytic fiber measurement in biologic samples and laboratories but stresses the need for laboratories to establish their own controls and to standardize selection criteria as well as methodology in controlled interlaboratory comparison studies.

Gylseth et al. (52) demonstrated marked interlaboratory variations in results using different techniques and instruments. Nevertheless, a trend to “high” versus “low” exposure could be provided by most institutions, suggesting that laboratories are reasonably consistent in detecting fiber content in asbestos-exposed individuals. De Vuyst et al. (16) concluded that the reference values (higher limit) for electron microscopic fiber counting in individuals without occupational asbestos exposure was roughly 1-2 × 10^6^ of total amphibole fibers and 0.1 × 10^6^ per gram dry lung tissue for amphibole fibers longer than 5 μm. A consensus proposed that subjects with a high probability of occupational asbestos exposure would be likely to have more than 1,000 asbestos bodies per gram of dry tissue, or for numbers of amphibole asbestos fibers longer than 1 μm exceed 1 × 10^6^/g dry tissue (>0.1 × 10^6^ for fibers longer than 5 μm) (25). Ranges for negative control subjects were not presented and each laboratory was advised to establish its own reference values. There can be little question that analytic laboratories should seek to focus on establishing their own internal controls for background non-disease subjects as well as for those with asbestosis.

Using the lower limit for high probability of occupational exposure, as proposed by the Helsinki criteria (23, 25) is intrinsically ambiguous and generates an overlap between control subjects and patients with occupational asbestos exposure being found in 23% of the reviewed studies (6/26). Thus, the use of structured questionnaires and checklists should be applied to identify and exclude subjects selected for control groups having undergone an occupational asbestos exposure (16, 23, 25). Adding to the complexity of separating control subjects from asbestos-exposed individuals, the decline in use and subsequent banning of asbestos in the western world between 1980 and 2010 has led to reduced occupational exposure resulting in lower lung tissue fiber content in patients with mesothelioma (27). Therefore, also ambient outdoor and indoor asbestos fiber concentrations have changed over the prior decades in developed Countries commensurate with the far more limited mining, manufacturing and end-product use of asbestos containing materials (53). Thus, it is plausible that fiber content in lung tissue from negative controls has also decreased over the last four decades. This trend appears when comparing the studies using SEM. And so controls established in analytic laboratories from past decades may not reflect actual environmental exposure in the post-ban period. Therefore, the authors recommend that analytic laboratories should seek to re-establish or update their control reference ranges, to reflect contemporaneous ambient asbestos exposures. The advent of advanced molecular genetics now allows individuals to undergo comprehensive genomic profiling, something which may be incorporated in controls as well as proposed disease cases, including mesothelioma, lung cancer and lung fibrosis. Future case control studies may determine that those subjects with inherited genetic syndromes (heterozygous carriers of pathogenic variant germline mutations), or those who harbor specific genetic polymorphisms in pro-inflammatory genes (TNF-a) or detoxification (GSTM1, GSTT1) have a modulated response to inhaled particulates which is presently not known or accounted for in published control studies.

It is also noted that some laboratories applied more rigorous multi-step inclusion criteria for their control selection, and these will be far less likely to be impacted by the passage of time. The characterization of appropriate controls for background subjects without disease has two clear implications—first in scientific risk-assessment exercises for the consideration of threshold models of exposure which have not been implicated in determining disease; second, in medicolegal settings, to appropriately benchmark an individual case exposure against a relevant control population. Establishing control reference ranges is not without considerable challenge given the difficulty in obtaining appropriate tissue samples. There is a recognized balance from use of surgical resections versus postmortem tissue. The former will inevitably result in more limited tissue availability, although comes with the advantage of potential first-hand characterization of exposures from the patient. The latter allows for widespread “pooled” biologic sample analysis, and more representative mineral content characterization. With respect to optimal tissue selection, the authors recommend the use of “pooled” lung tissue samples which incorporate different anatomic tissue locations and allow for the most representative results of an individual’s prior exposure as already outlined by the guidelines (16, 24). The use of micro-dissected pleural strips, anthracotic lymph nodes, omentum, mesenterial or tumor-containing tissue is not advocated for routine use. The review by Caraballo-Arias and co-authors (54) on pleural tissue showed that case numbers in general are small and controls without asbestos-related disease often missing. The study did not make clear, however, that analytic epidemiology correlates asbestos-related diseases with retained asbestos fibers in pleura. The authors mention several possible complications occurring in analyzing pleura and stress the need for further research. The individual preference is laboratory based although it is clearly preferential to establish controls in tissue most commonly handled by an individual laboratory for test case analyses to maintain a consistency in analytic handling.

Asbestos content of control groups may be expressed as ranges of values if individuals are compared to a group. If two groups (e.g., control vs. exposed group) are compared generally mean and standard deviation are used. Because fiber counts represent not-normally distributed data numbers of fibers should rather be expressed as median and quartile in this context. Reporting range as well as median allows for different usage of data.

It is clear that the observed lack of methodologic standardization across analytic laboratories may be enhanced to establish more robust controls by a number of proposals, which include (1) Establishing an International Task-Force Working Group for the Analysis of Biologic Samples which effectively sets out a consensus methodology for sample preparation, fiber counting and result validation in biologic samples; (2) Establish a robust consensus by determining strict inclusion criteria for control cases. To this end, it is recognized selection bias of cases with remote occupational, para-occupational and environmental/endemic may influence reference cases and upper bound exposure limits. The incorporation of exposure questionnaires is useful in this regard to exclude such cases; (3) establish a network of analytic laboratories engaging in inter-laboratory case control testing, specimen exchange and same sample analysis, with peer-review examination of the variability of results, and proposal and adoption of an external quality audit program. (4) Future plans may incorporate the integration of comprehensive genomic profiling to research for potential individual susceptibility to inhaled particulates, and to correlate this with extent of disease. (5) Implement a longitudinal study in populations with fully characterized exposure data to a wide array of minerals to examine for potential interactions and effects of habits, e.g., tobacco smoking.

In summary, control background groups hold a profound significance in determining the relevance of exposure and disease. They are essential to all analytical laboratories to achieve valid and reliable results. These groups make it possible to verify the causal relationship between asbestos exposure and the subsequent diseases, to avoid distortions and to evaluate the effectiveness of prevention and treatment options. To optimize identification of negative control subjects we propose to use the following criteria: (1) exclusion of occupational, para-occupational, and environmental history of asbestos exposure using structured questionnaires, (2) in autopsy cases death should not be related to asbestos, (3) absence of pleural plaques, and (4) absence of asbestos bodies in sections by light microscopy. Additionally, if possible, controls should be updated periodically, maybe each decade. In addition, control subjects should be matched according to age and gender. To improve comparison of studies, fiber dimensions (e.g., length > 5 μm and width < 0.25 μm) should be specified and fiber counts expressed as median and range.

The analysis of fiber burden in the lung tissue of control subjects, who have been exposed to ambient air, serves as a baseline for understanding environmental exposure to airborne fibers. This baseline is critical for comparing lung fiber burden in occupationally exposed individuals or those with specific environmental exposures. Given the potential long-term consequences of asbestos exposure, it is crucial that future studies in this area continue to use strictly controlled methods and negative controls to better understand and mitigate the health risks of asbestos exposure.

## 5 Conclusion

Mineral fiber analysis represents the best arbiter of determining an individual’s prior exposures to respirable asbestos fibers which correlate with disease. An appreciation of controls in analytical laboratories allows researchers to contextualize the significance of fiber burdens and dimensions in individual cases. Due to large differences in geological environments, degree of urbanization and industrialization between populations as well as different methods used in various laboratories control groups should be established for each laboratory within the local setting and using local methods. All analytic laboratories are required to establish their own control reference ranges for subjects without disease or occupational exposure, as well as those with disease – asbestosis, in the setting of the asbestosis range for lung cancer attribution and for the establishment of asbestosis. Ambient asbestos fiber concentrations are subject to change with time, especially in Countries in which asbestos is banned. Accordingly control reference populations should be periodically updated to reflect these lowering exposures so that controls are contemporaneous to periods when testing is conducted in cases. It would be wise to report median and range for asbestos body and fiber content as it allows a range of applications. All analytic laboratories evaluating asbestos fiber content in biologic samples require intra- and interlaboratory standardization to seek to address the issues of quality, validation and accurate mineralogic characterization.

## Data Availability

The original contributions presented in the study are included in the article/supplementary material, further inquiries can be directed to the corresponding authors.

## References

[1] Whitwell F Scott J Grimshaw M. Relationship between occupations and asbestos fibre content of the lungs in patients with pleural mesothelioma, lung cancer, and other diseases. Thorax. (1977) 32:377–86. doi: 10.1136/thx.32.4.377, PMID: 929482 PMC470635

[2] Churg AM Warnock ML. Asbestos and other ferruginous bodies. Their formation and clinical significance. Am J Pathol. (1981) 102:447–56. PMID: 6101235 PMC1903711

[3] Wagner JC Sleggs CA Marchand P. Diffuse pleural mesothelioma and asbestos exposure in the North Western Cape Province. Br J Ind Med. (1960) 17:260–71. doi: 10.1136/oem.17.4.260, PMID: 13782506 PMC1038078

[4] Wagner JC Moncrieff CB Coles R Griffiths DM Munday DE. Correlation between fibre content of the lungs and disease in naval dockyard workers. Br J Ind Med. (1986) 43:391–5. doi: 10.1136/oem.43.6.391, PMID: 3718883 PMC1007669

[5] Wagner JC Newhouse ML Corrin B Rossiter CER Griffiths DM. Correlation between Fiber content of the lung and disease in East London Asbestos factory-workers. Br J Ind Med. (1988) 45:305–8. PMID: 3378009 10.1136/oem.45.5.305 PMC1007999

[6] Churg A Vedal S. Fiber burden and patterns of asbestos-related disease in workers with heavy mixed amosite and chrysotile exposure. Am J Respir Crit Care Med. (1994) 150:663–9. doi: 10.1164/ajrccm.150.3.8087335, PMID: 8087335

[7] Roggli VL Oury TD Sporn TA. Pathology of Asbestos-associated diseases, Second Edition. New York: Springer; (2004). 1–437 p.

[8] ATSDR. Toxicological profile for Asbestos. Atlanta: Agency for Toxic Substances and Disease Registry (2001). 37983317

[9] WHO/IARC. Asbestos, summary of data reported and evaluation. Lyon: WHO/IARC (1998). 14 p.

[10] Price B Ware A. Epidemiology trends in the United States: an update based on surveillance, epidemiology, and end results program data for 1973 through 2003. Am J Epidemiol. (2004) 159:107–12. doi: 10.1093/aje/kwh025, PMID: 14718210

[11] Teta MJ Mink PJ Lau E Sceurman BK Foster ED. US mesothelioma patterns 1973-2002: indicators of change and insights into background rates. Eur J Cancer Prev. (2008) 17:525–34. doi: 10.1097/CEJ.0b013e3282f0c0a2, PMID: 18941374

[12] Glynn ME Keeton KA Gaffney SH Sahmel J. Ambient Asbestos Fiber concentrations and long-term trends in pleural mesothelioma incidence between urban and rural areas in the United States (1973-2012). Risk Anal. (2018) 38:454–71. doi: 10.1111/risa.12887, PMID: 28863229

[13] Keeton KA Glynn ME Gaffney SH Sahmel J. Response to letter to the editor regarding “ambient Asbestos Fiber concentrations and long-term trends in pleural mesothelioma incidence between urban and rural areas in the United States (1973-2012)” by Finkelstein. Risk Anal. (2018) 38:1524–8. doi: 10.1111/risa.13169, PMID: 30133854

[14] Mowe G Gylseth B Hartveit F Skaug V. Occupational Asbestos exposure, lung-Fiber concentration and latency time in malignant mesothelioma. Scand J Work Environ Health. (1984) 10:293–8. doi: 10.5271/sjweh.2326, PMID: 6523093

[15] Srebro SH Roggli VL Samsa GP. Malignant mesothelioma associated with low pulmonary tissue asbestos burden: a light and scanning electron microscopic analysis of 18 cases. Mod Pathol. (1995) 8:614–21. PMID: 8532693

[16] De Vuyst P Karjalainen A Dumortier P Pairon JC Monso E Brochard P. Guidelines for mineral fibre analyses in biological samples: report of the ERS working group. Eur Respir J. (1998) 11:1416–26. doi: 10.1183/09031936.98.11061416, PMID: 9657589

[17] Dodson RF Williams MG Huang J Bruce JR. Tissue burden of asbestos in nonoccupationally exposed individuals from East Texas. Am J Ind Med. (1999) 35:281–6. doi: 10.1002/(SICI)1097-0274(199903)35:3<281::AID-AJIM8>3.0.CO;2-O, PMID: 9987561

[18] Gibbs AR Pooley FD Attanoos RL. Establishing ‘control’standards to aid the diagnosis of asbestosis; asbestos fibre burden and fibrosis in the lungs of non-occupationally exposed persons. Lab Investig. (2005) 85:311a–a.

[19] Roggli VL. The so-called short-Fiber controversy literature review and critical analysis. Arch Pathol Lab Med. (2015) 139:1052–7. doi: 10.5858/arpa.2014-0466-RA, PMID: 26230599

[20] Attanoos RL Alchmi F Gibbs AR. A correlative analysis of Asbestos fibers, bodies, fibrosis, and controls: predictions on mineral analysis from tissue sections. Arch Pathol Lab Med. (2016) 140:262.

[21] Attanoos RL Churg A Galateau-Salle F Gibbs AR Roggli VL. Letter to the editor re: mesothelioma and its non-asbestos causes—spontaneous mesothelioma in women. Arch Pathol Lab Med. (2019) 143:911–914. 31339754 10.5858/arpa.2019-0060-LE

[22] Roggli VL Sharma A Butnor KJ Sporn T Vollmer RT. Malignant mesothelioma and occupational exposure to asbestos: a clinicopathological correlation of 1445 cases. Ultrastruct Pathol. (2002) 26:55–65. doi: 10.1080/01913120252959227, PMID: 12036093

[23] Tossavainen A. Asbestos, asbestosis, and cancer: the Helsinki criteria for diagnosis and attribution. Scand J Work Env Heal. (1997) 23:311–6. doi: 10.5271/sjweh.226 9322824

[24] Roggli VL Gibbs AR Attanoos R Churg A Popper H Cagle P. Pathology of asbestosis-an update of the diagnostic criteria report of the asbestosis Committee of the College of American pathologists and pulmonary pathology society. Arch Pathol Lab Med. (2010) 134:462–80. doi: 10.5858/134.3.462, PMID: 20196674

[25] Wolff H Vehmas T Oksa P Rantanen J Vainio H. Asbestos, asbestosis, and cancer, the Helsinki criteria for diagnosis and attribution 2014: recommendations. Scand J Work Env Heal. (2015) 41:5–15. doi: 10.5271/sjweh.3462, PMID: 25299403

[26] Roggli VL Vollmer RT. Twenty-five years of fiber analysis: what have we learned? Hum Pathol. (2008) 39:307–15. doi: 10.1016/j.humpath.2007.07.005, PMID: 18187182

[27] Roggli VL Green CL Liu B Carney JM Glass CH Pavlisko EN. Chronological trends in the causation of malignant mesothelioma: Fiber burden analysis of 619 cases over four decades. Environ Res. (2023) 230:114530. doi: 10.1016/j.envres.2022.114530, PMID: 36965800 PMC10542945

[28] Attanoos RL Churg A Galateau-Salle F Gibbs AR Roggli VL. Malignant Mesothelioma and Its Non-Asbestos Causes. Arch Pathol Lab Med. (2018) 142:753–60. doi: 10.5858/arpa.2017-0365-RA 29480760

[29] Attanoos RL Gibbs AR. Pathology of malignant mesothelioma. Histopathology. (1997) 30:403–18. doi: 10.1046/j.1365-2559.1997.5460776.x 9181361

[30] IARC (ed.). Asbestos (chrysotile, amosite, crocidolite, tremolite, actinolite, and anthophyllite) In: IARC monographs on the evaluation of carcinogenic risks to humans a review in human carcinogens part C: arsenic, metals, fibres, and dusts. Lyon: World Health Organization (2012). 219–309. PMC4781271 23189751

[31] Price B. Projection of future numbers of mesothelioma cases in the US and the increasing prevalence of background cases: an update based on SEER data for 1975 through 2018. Crit Rev Toxicol. (2022) 52:317–24. doi: 10.1080/10408444.2022.2082919, PMID: 35852497

[32] Carbone M Arron ST Beutler B Bononi A Cavenee W Cleaver JE. Tumour predisposition and cancer syndromes as models to study gene–environment interactions. Nat Rev Cancer. (2020) 20:533–49. doi: 10.1038/s41568-020-0265-y, PMID: 32472073 PMC8104546

[33] Carbone M Pass HI Ak G Alexander HR Baas P Baumann F. Medical and surgical Care of Patients with Mesothelioma and Their Relatives Carrying Germline BAP1 mutations. J Thorac Oncol. (2022) 17:873–89. doi: 10.1016/j.jtho.2022.03.014, PMID: 35462085 PMC9233126

[34] Roggli VL Pratt PC Brody AR. Asbestos content of lung tissue in asbestos associated diseases: a study of 110 cases. Br J Ind Med. (1986) 43:18–28. doi: 10.1136/oem.43.1.18, PMID: 3947558 PMC1007596

[35] Case BW Sebastien P. Environmental and occupational exposures to chrysotile asbestos: a comparative microanalytic study. Arch Environ Health. (1987) 42:185–91. PMID: 2821933

[36] Gaudichet A Janson X Monchaux G Dufour G Sebastien P De Lajartre AY. Assessment by analytical microscopy of the total lung fibre burden in mesothelioma patients matched with four other pathological. Ann Occup Hyg. (1988) 32:213–23. doi: 10.1016/B978-0-08-034185-9.50027-9, PMID: 40748484

[37] Albin M Johansson L Pooley FD Jakobsson K Attewell R Mitha R. Mineral fibres, fibrosis, and asbestos bodies in lung tissue from deceased asbestos cement workers. Br J Ind Med. (1990) 47:767–74. doi: 10.1136/oem.47.11.767, PMID: 2173948 PMC1035268

[38] Romer M. Präparation und mineralogische Analytik von Lungenstaub. Zurich: ETH Zurich (1993).

[39] Dawson A Gibbs AR Pooley FD Griffiths DM Hoy J. Malignant mesothelioma in women. Thorax. (1993) 48:269–74. doi: 10.1136/thx.48.3.269, PMID: 8497827 PMC464367

[40] Sakai K Hisanaga N Huang J Shibata E Ono Y Aoki T. Asbestos and nonasbestos fiber content in lung tissue of Japanese patients with malignant mesothelioma. Cancer. (1994) 73:1825–35. doi: 10.1002/1097-0142(19940401)73:7<1825::AID-CNCR2820730709>3.0.CO;2-M, PMID: 8137206

[41] Dodson RF Huang J Bruce JR. Asbestos content in the lymph nodes of nonoccupationally exposed individuals. Am J Ind Med. (2000) 37:169–74. doi: 10.1002/(SICI)1097-0274(200002)37:2<169::AID-AJIM2>3.0.CO;2-V, PMID: 10615097

[42] Roggli VL Sharma A. Analysis of mineral content In: Roggli VL Oury TD Sporn TA, editors. Pathology of Asbestos-associated diseases, second edition. 2nd ed. New York: Springer (2004). 309–54.

[43] Han JH Park JD Sakai K Hisanaga N Chang HK Lee YH. Comparison of lung Asbestos Fiber content in Cancer subjects with healthy individuals with no known history of occupational Asbestos exposure in Korea. J Toxicol Environ Heal. (2009) 72:1292–5. doi: 10.1080/15287390903212345, PMID: 20077199

[44] Gilham C Rake C Burdett G Nicholson AG Davison L Franchini A. Pleural mesothelioma and lung cancer risks in relation to occupational history and asbestos lung burden. Occup Environ Med. (2016) 73:290–9. doi: 10.1136/oemed-2015-103074, PMID: 26715106 PMC4853597

[45] Velasco-Garcia MI Cruz MJ Diego C Montero MA Alvarez-Simon D Ferrer J. First identification of pulmonary Asbestos Fibres in a Spanish population. Lung. (2017) 195:671–7. doi: 10.1007/s00408-017-0042-1, PMID: 28791466

[46] Gordon RE. Analytical analyses of human tissues for the presence of Asbestos and talc. Electron Microsc Nov Microsc Trends. (2019) 10:1–19. doi: 10.5772/intechopen.83656

[47] Schneider J Arhelger R Brückel B Baur X. Diagnostic limitations of lung fiber counts in asbestos-related diseases. J Sci Pract Integr. (2023) 1–16. doi: 10.35122/001c.70352, PMID: 29248759

[48] Visonà SD Bertoglio B Favaron C Capella S Belluso E Colosio C. A postmortem case control study of asbestos burden in lungs of malignant mesothelioma cases. J Transl Med. (2023) 21:1–10. doi: 10.1186/s12967-023-04761-9, PMID: 38041166 PMC10693031

[49] Churg A Warnock ML. Asbestos fibers in the general population. Am Rev Respir Dis. (1980) 122:669–78. doi: 10.1164/arrd.1980.122.5.669, PMID: 7447151

[50] Churg A. Fiber counting and analysis in the diagnosis of asbestos-related disease. Hum Pathol. (1982) 13:381–92. doi: 10.1016/S0046-8177(82)80227-X, PMID: 6281166

[51] Mowé G Gylseth B Hartveit F Skaug V. Fiber concentration in lung tissue of patients with malignant mesothelioma a case—control study. Cancer. (1985) 56:1089–93. doi: 10.1002/1097-0142(19850901)56:5<1089::AID-CNCR2820560522>3.0.CO;2-Y, PMID: 4016698

[52] Gylseth B Churg A Davis JMGG Johnson N Morgan A Mowe G. Analysis of Asbestos fibers and Asbestos bodies in tissue samples from human-lung—an international Interlaboratory trial. Scand J Work Environ Health. (1985) 11:107–10. doi: 10.5271/sjweh.2246, PMID: 4001898

[53] Abelmann A Glynn ME Pierce JS Scott PK Serrano S Paustenbach DJ. Historical ambient airborne asbestos concentrations in the United States—an analysis of published and unpublished literature (1960s-2000s). Inhal Toxicol. (2015) 27:754–66. doi: 10.3109/08958378.2015.1118172, PMID: 26671195

[54] Caraballo-Arias Y Caffaro P Boffetta P Violante FS. Quantitative assessment of Asbestos fibers in Normal and pathological pleural tissue—a scoping review. Life. (2022) 12:1–12. doi: 10.3390/life12020296, PMID: 35207583 PMC8878760

